# Sensorimotor predictions shape reported conscious visual experience in a breaking continuous flash suppression task

**DOI:** 10.1093/nc/niab003

**Published:** 2021-03-18

**Authors:** Lina I Skora, Anil K Seth, Ryan B Scott

**Affiliations:** School of Psychology, University of Sussex, Pevensey Building, Falmer, Brighton BN1 9RH, UK; Sackler Centre for Consciousness Science, University of Sussex, Falmer, Brighton, Brighton BN1 9RH, UK; Sackler Centre for Consciousness Science, University of Sussex, Falmer, Brighton, Brighton BN1 9RH, UK; School of Engineering and Informatics, University of Sussex, Falmer, Brighton, Brighton BN1 9RH, UK; Canadian Institute for Advanced Research, Program on Brain, Mind and Consciousness, 661 University Ave, Toronto, ON M5G 1M1, Canada; School of Psychology, University of Sussex, Pevensey Building, Falmer, Brighton BN1 9RH, UK; Sackler Centre for Consciousness Science, University of Sussex, Falmer, Brighton, Brighton BN1 9RH, UK

**Keywords:** consciousness, perception, unconscious processing, continuous flash suppression, breaking-CFS

## Abstract

Accounts of predictive processing propose that conscious experience is influenced not only by passive predictions about the world, but also by predictions encompassing how the world changes in relation to our actions—that is, on predictions about sensorimotor contingencies. We tested whether valid sensorimotor predictions, in particular learned associations between stimuli and actions, shape reports about conscious visual experience. Two experiments used instrumental conditioning to build sensorimotor predictions linking different stimuli with distinct actions. Conditioning was followed by a breaking continuous flash suppression task, measuring the speed of reported breakthrough for different pairings between the stimuli and prepared actions, comparing those congruent and incongruent with the trained sensorimotor predictions. In Experiment 1, counterbalancing of the response actions within the breaking continuous flash suppression task was achieved by repeating the same action within each block but having them differ across the two blocks. Experiment 2 sought to increase the predictive salience of the actions by avoiding the repetition within blocks. In Experiment 1, breakthrough times were numerically shorter for congruent than incongruent pairings, but Bayesian analysis supported the null hypothesis of no influence from the sensorimotor predictions. In Experiment 2, reported conscious perception was significantly faster for congruent than for incongruent pairings. A meta-analytic Bayes factor combining the two experiments confirmed this effect. Altogether, we provide evidence for a key implication of the action-oriented predictive processing approach to conscious perception, namely that sensorimotor predictions shape our conscious experience of the world.

## Introduction

A growing body of experimental work, rooted in the predictive processing framework (Rao and Ballard, 1999; Clark, 2013; Hohwy, 2013, 2020), shows that perceptual experiences are influenced by beliefs or predictions about the world. Valid predictions have been shown to facilitate access to visual consciousness (Melloni *et al.*, 2011; Pinto *et al.*, 2015; De Loof *et al.*, 2016; Meijs *et al.*, 2018,b), reduce repetition suppression (Summerfield *et al.*, 2008), improve metacognition (Sherman *et al.*, 2015), and aid interpretation under perceptual ambiguity (Panichello *et al.*, 2013; Aru *et al.*, 2016).

Within the predictive processing framework, predictions are instantiated by probabilistic generative models, encoded in cortical hierarchies. Incoming sensory signals, such as visual input, are compared against descending predictions to give rise to prediction errors (PEs) at each hierarchical level of processing. Minimization of PEs across hierarchical levels implements an approximation to Bayesian inference on the causes of sensory signals. In this framework, conscious sensory experience has been proposed to reflect the perceptual prediction that best suppresses PEs (e.g. Hohwy, 2013; Seth *et al.*, 2016). In other words, conscious experience is shaped, or constituted, by the posterior prediction that ‘best’ predicts the (hidden) causes of sensory signals.

Importantly, minimization of PEs can occur both by updating predictions and by performing actions that (are predicted to) furnish predicted sensory data. PE minimization through action is known as ‘active inference’ (Friston *et al.*, 2010). From an active inference perspective, perceptual experience is influenced not only by ‘passive’ predictions about the world, but more generally by predictions encompassing the coupling or contingency between actions and sensory signals—i.e. on predictions about sensorimotor contingencies (O’Regan and Noë, 2001; Seth, 2014; Clark, 2015). According to this view, the ‘winning’ predictions are not necessarily those which are the most veridical, but those which best support adaptive interactions with the world (Seth, 2014, 2015; Clark, 2015, 2016; Tschantz *et al.*, 2020). Action is therefore not just an ‘output’ that follows perceptual inference, but is an integral part of our experience of the world.

How do predictions about sensorimotor contingencies affect perceptual experience? Proponents of active inference argue that action execution leads to a generalized ‘sensory attenuation’ across all modalities (Brown *et al.*, 2013), such that sensitivity to all sensory events is reduced during action. However, this leaves open the important question of how conscious perception is shaped by the validity of predictions about sensorimotor contingencies, where such contingencies could reflect, for example, learned associations between a stimulus and an action?

On one view, in line with the facilitatory effects of ‘passive’ perceptual predictions mentioned above, valid predictions about sensorimotor contingencies should enhance perception. Evidence supporting this comes from studies showing that action can help disambiguate a bistable or otherwise ambiguous percept if it is congruent with an aspect of that percept, for example when the direction of movement corresponds to the direction of moving dots (Maruya *et al.*, 2007; Mitsumatsu, 2009; Beets *et al.*, 2010; Di Pace and Saracini, 2014; Suzuki *et al.*, 2019). Here, action is interpreted as providing a predictive cue, biasing or sharpening action-congruent percepts (Yon et al., 2018, 2020a,b; Press *et al.*, 2020).

On another view, valid predictions should lead to weaker perception, on the logic that the corresponding sensory data will be ‘cancelled out’ by the congruent prediction (Wolpert *et al.*, 1995; Bays and Wolpert, 2007). Evidence for this view has been provided predominantly in the domain of touch (famously illustrated by our inability to tickle ourselves, Blakemore *et al.*, 1998). However, some studies suggest that perception of action-congruent outcomes in visual perception may also be attenuated (Cardoso-Leite *et al.*, 2010; but see Schwarz *et al.*, 2018).

Another important issue is the origin of the predictions about sensorimotor contingencies. In many of the studies reported, valid predictions likely reflect long-term structural learning—such as learning which movement will result in a specific direction of object motion (Maruya *et al.*, 2007; Dogge *et al.*, 2019)––rather than the fluid, context-dependent expectations that are highlighted within predictive processing and active inference.

Here, we set out to test whether predictions about sensorimotor contingencies affect reportable conscious perception of visual stimuli. We did this by training arbitrary, contextual (as opposed to structural) stimulus-action associations, and then examining the contribution to conscious experience made by the congruency of sensorimotor predictions. Importantly, we interpret ‘congruency with sensorimotor predictions’ in terms of the action a person takes *in response to* a stimulus, as opposed to the notion of ‘stimulus-action’ congruence in which, for example, there is a mere correspondence between the direction of an agent’s action and the direction of some stimuli in the world.

We developed a novel two-stage paradigm in which we operationalized sensorimotor contingencies as learned arbitrary associations between visual stimuli and subsequent actions. We call these learned associations ‘sensorimotor predictions’. To examine the effects of valid versus invalid sensorimotor predictions on speed of access to visual consciousness, we used the proxy of breakthrough time in continuous flash suppression (b-CFS; Tsuchiya and Koch, 2005; Jiang *et al.*, 2007); we return to the limitations of b-CFS for measuring the speed of conscious access later.

In the first stage of the paradigm, we used instrumental conditioning to build sensorimotor predictions by linking distinct stimuli with specific actions. Two stimuli were arbitrarily associated with equally simple but distinguishable actions (an index finger or a little finger button press), and a third stimulus was associated with no action. In this design, actions are made in response to stimuli (in line with stimulus-response conditioning paradigms), rather than actions triggering a stimulus or causing some other change in the world. Thus, in a sense, we are building sensorimotor predictions in a ‘reversed’ direction (Elsner and Hommel, 2001, 2004)––an issue we return to in the Discussion.

In the second stage, each of the three stimuli used in the conditioning task, as well as a fourth, novel, previously unseen stimulus, was presented under CFS, where a dynamically flashing high-contrast pattern displayed to one eye is used to suppress visual awareness of the target stimulus displayed to the other eye, while participants prepared to respond with stimulus-congruent or non-congruent action. We were therefore able to examine the extent to which maintaining a valid prediction of a sensorimotor association (through response preparation) facilitates reported conscious access to the associated stimulus, compared to an invalid prediction. Speed of access of each stimulus to visual consciousness was assessed using breakthrough time in CFS (breaking-CFS or b-CFS; Jiang *et al.*, 2007), measuring the time it takes for the target to overcome interocular suppression and become consciously visible. We hypothesised that preparing an action associated through training with a specific stimulus should engage a valid sensorimotor prediction, facilitating conscious experience of that stimulus and yielding faster reported breakthrough times.

Two experiments were conducted with the same conditioning task but with minor differences to the b-CFS task. In Experiment 1, the counterbalancing of action and stimuli was achieved at the block level by having the same prepared action for each trial within a b-CFS block, with actions differing only between blocks. As each block contained one presentation of each of four visual stimuli, this ensured that each action was paired with each stimulus on only one occasion. For example, if the action for block 1 was the index finger button press, participants would use their index finger to respond when each of the four different visual stimuli broke through b-CFS. Then in block 2, they would use their little finger to respond, again as each of the four different visual stimuli broke through b-CFS. However, a limitation of this design is that enforcing repetition of one action within a block may have reduced that action’s predictive salience. Experiment 2 addressed this limitation by varying the prepared actions within each b-CFS block, while still ensuring counterbalancing of the stimulus-action pairing across the two blocks. A second minor variation made to the b-CFS task in Experiment 2 sought to separate two different contributions to response times. In Experiment 1 participants had to indicate the orientation of a single pixel line overlaid on the stimulus at the moment it broke through suppression; the inclusion of this judgment seeks to reduce premature responses. In Experiment 2, this line orientation judgment was made subsequent to the initial reaction time response, thus avoiding adding noise to the measure of breakthrough time. Both Experiments were pre-registered on the Open Science Framework at https://osf.io/ensba (Experiment 1) and https://osf.io/ez62m (Experiment 2). All material, task code and data are available at https://osf.io/hpsju/.

## Experiment 1

### Materials and Methods

#### Participants

68 participants (11 male; *M* age = 20, *SD* = 2.38, range: 19–32) were recruited for the study via the University of Sussex online recruitment system, and an internal mailing list. All participated in exchange for course credit. Participants were required to have normal or corrected-to-normal vision, and no current or history of neurological illness. Ethical approval was granted by the Science and Technology Cross-School Research Ethics Committee at the University of Sussex, and the study was conducted in accordance with the Declaration of Helsinki.

#### Stimuli and materials

The experiment was implemented in Matlab 2017 b (MathWorks, 2017) with the Cogent2000 toolbox (UCL LoN, 2003). All stimuli were presented on a Dell monitor (1280 x 1024) with a refresh rate of 60 Hz. Responses were collected with a standard keyboard.

**Figure 1.**
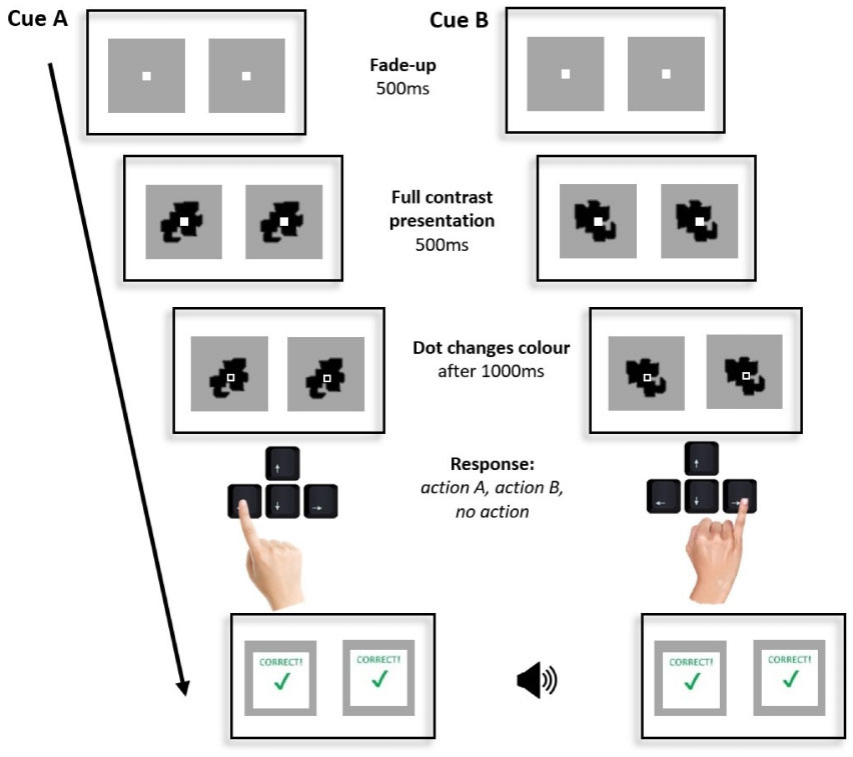
Instrumental conditioning task. Chronological screenshots depict a single trial sequence for the action cues—each panel shows the images shown to the left and right eyes. After 1000 ms (including 500 ms fade-up of the cue), the fixation dot changes colour from white to black (with a white border), and participants can execute the desired action (A, an index finger button press on the left arrow, or B, a little finger button press on the right arrow). For a no-action cue, the fixation dot would change colour from red to blue. Here, the action executed corresponded to the cue type, and the participant was rewarded with visual and auditory feedback.

**Figure 2.**
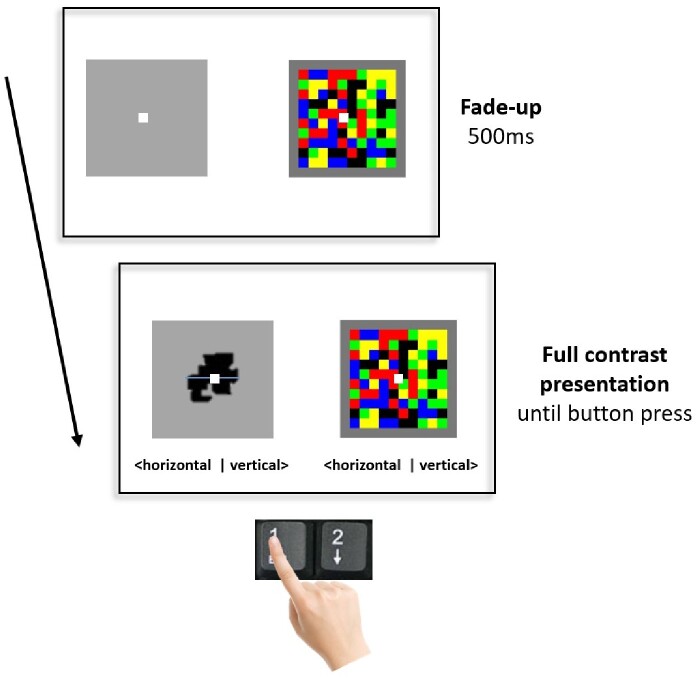
Breaking-CFS task. Screenshots depict a single trial sequence (identical for all cues). Following 500 ms fade-up, the cue remained on-screen with a horizontal or vertical line overlaid on top of it. Participants were requested to make a response indicating the observed line orientation as soon as they could. This response was to be made with either their index finger or little finger depending on the action randomly assigned to that block. On a given trial, the cue presented could be congruent with the trained action (i.e. associated with it in the conditioning stage), incongruent with it, associated with no action, or novel (not associated with any action).

The target stimuli included three sets of four cues. Each set contained four 90^°^ rotations of the same symbol—a neutral, asymmetrical shape, generated by manually overlaying shapes on each other to generate an abstract figure which looks distinct upon rotation (e.g. Fig. 1). All stimuli were 119 by 119 pixels in size (3.43^°^ visual angle), presented in dark grey (RGB: 80,80,80) on lighter grey (RGB: 128,128,128) background. The Mondrian patterns for CFS were composed of coloured rectangles in a box 240 by 240 pixels in size (6.91^°^ visual angle; Fig. 2). On each trial, 10 different Mondrian patterns were presented in a random non-repeating sequence every second (i.e. the pattern changed at 10 Hz).

**Figure 3.**
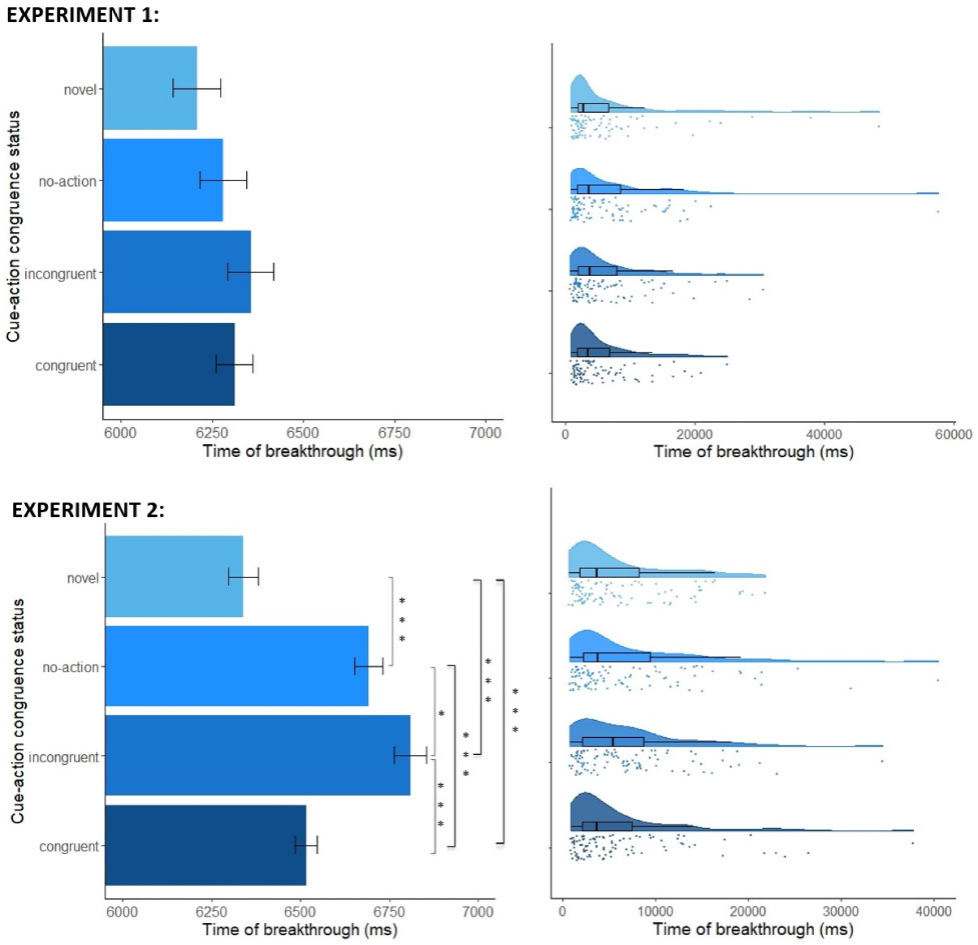
Left panels: Mean reported times of breakthrough (ms) by cue-action congruence status in Experiment 1 (top; *N* = 49) and Experiment 2 (bottom; *N* = 55), estimated by the GLMM (+/– 1 SEM). Stars indicate *P *<* *0.05. Right panels: Raw distributions of breakthrough times with boxplots in Experiments 1 and 2. Plots created following Allen *et al.* (2019).

#### Procedure

Each participant completed two experimental conditions in a single experimental session, one where conditioning was completed consciously (where stimuli were clearly visible), and a second where conditioning was attempted subliminally (where stimuli were presented without conscious awareness). The conditions consisted of the same two main stages. Stage 1 was an instrumental conditioning task (2 blocks of 60 trials), and Stage 2 was a breaking-CFS task (2 blocks of 4 trials each). A brief set of practice trials was completed prior to Stage 1. Throughout both stages, participants were asked to look at the screen through a mirror stereoscope, fitted atop a chinrest at a 50 cm distance from the screen. Ocular dominance was established prior to beginning the experiment with a standard Miles test (Miles, 1930).

Here, we report the conscious condition only, as we found strong evidence that the stimulus-action associations were not learnt in the unconscious condition (see Supplementary Material for the procedure and results of the unconscious instrumental conditioning task). Given that our primary interest here is to examine the effect that predictions about learned stimulus-action associations have on breaking-CFS, it would not be informative to analyse data from a condition where those associations were not acquired. The order of the conditions was randomized for each participant. The conditions were independent, and used different sets of stimuli (randomized).

##### Conditioning task

The conditioning task was used to establish the sensorimotor (stimulus-action) associations. The task used three stimuli selected from the assigned set of four. One of the stimuli (*cue A*) was paired with action A (an index finger button press), a second (*cue B*) with action B (a little finger button press), and a third (*no-action cue*) with no action. For practical reasons, the index finger button press was made on the left arrow, and the little finger button press on the right arrow. Note that while the keys being pressed differed, we consider the finger used to make the button press to be the conditioned action, not the button. Cues were randomly assigned to a given stimulus type for each participant. The order of presentation was randomized with an equal number of exposures to each cue occurring in each block of trials. Participants performed 120 conditioning trials in two blocks of 60, with a 1 min break between the blocks.

In this task, both eyes were presented with the stimuli, i.e. there was no CFS and the stimuli were clearly visible. On each trial, stimulus presentation started with a fade-up period of 500 ms. A further 500 ms after the stimulus reached full contrast, the fixation dot (overlaid on top of the cue) changed colour, indicating that a response was required (see Fig. 1 for a trial sequence). Fixation dot colours provided the distinction between action and no-action trials. For action cues, participants were instructed to respond with either action A or B (of their choosing), when prompted by the fixation dot changing colour from white to black. Following the action, positive or negative reinforcing feedback was delivered (‘correct!’ or ‘wrong!’ printed on the screen paired with a cash register or buzz sound, respectively) depending on the correspondence between the executed action and the cue presented, thus instantiating instrumental conditioning. For example, if *cue A* was presented and *action A* was executed, positive feedback was delivered, but if cue A was presented and *action B* was executed, negative feedback was delivered. If a participant refrained from action in response to an action cue, they received negative feedback. For *no-action cues*, the fixation dot started as blue, and changed colour to red. This indicated to participants at the onset of the trial that no response will be required. In these no-action trials negative feedback was delivered if participants responded with either action A or B, and positive feedback if they correctly refrained from action. As such, the presented cue became associated with the ‘no action’ response.

On action trials, the cue disappeared as soon as participants made a response (a total of 1000 ms + reaction time after cue onset). This ensured that the action was prepared and executed while the participant was exposed to the cue. On no-action trials, the cue disappeared as soon as the fixation dot changed which for these trials was set to be 1500 ms after cue onset (500 ms longer than in action trials). This additional 500 ms sought to roughly equalize the total exposure duration for no-action trials, where there was no response time, and action trials where the exposure extended to include the participant’s response time.

##### Breakthrough task

The conditioning task was immediately followed by a breaking-CFS task, using the set of cues from the conditioning task (*cue A, cue B, no-action cue)*, as well as the fourth, novel cue, never previously seen (the last from the set). In the breakthrough task, the stimuli were presented under CFS, with the target cue presented to the non-dominant eye, while the dominant eye received a Mondrian pattern (see *Stimuli and materials* Section).

Each cue was presented with a randomly assigned white horizontal or vertical line (1 pixel wide) overlaid on top of it. In order to quantify the time of breakthrough, participants were asked to report the orientation of the line using the ‘1’ (horizontal) and ‘2’ (vertical) buttons on the keypad, as soon as they were able to discriminate it. Note that they were required to report the line orientation even for cues previously associated with ‘no-action’. Despite the fact that the line orientation judgment was not part of the stimulus-action congruency training, we used it to quantify the time of breakthrough, reasoning that enhanced perception of the dark grey stimulus should be accompanied by enhanced perception of the white line overlaid on top of it. On each trial, the target cue faded-up over 500 ms, and remained on screen in full contrast, concurrently with the continuously flashing Mondrian pattern, until the response was made. See Fig. 2 for an illustration of the trial sequence.

The task was split into two blocks, with all four stimuli presented in each block in a randomized order, resulting in a total of eight trials. When indicating the line orientation, participants were required to make their response with the same action for all the trials in a single block, i.e. using the index finger to press button ‘1’ or ‘2’, or the little finger to press button ‘1’ or ‘2’ throughout. The assigned action (use of index finger or little finger) was randomized between blocks. This design ensured that each cue was matched with each action type once (e.g. action A is performed to indicate time of breakthrough for cue A, cue B, no-action cue, and the novel cue in one block, and action B in the other).

### Results

#### Bayes factors

For hypothesis testing, Bayes Factors (Bs) will be reported alongside *P*-values for all comparisons. Bs can help to disambiguate non-significant results as either indicating support for the null hypothesis (H_0_, positing no effect), support for the alternative hypothesis (H_1_, which uses an estimated raw effect size as the standard deviation of its distribution), or indicating insensitive data (i.e. the data are not in favour of either H_0_ or H_1_; Dienes, 2014). By convention, Bs smaller than 1/3 indicate evidence for H_0_. Bs larger than 3 indicate evidence for H_1_. Bs between those values indicate insensitive data.

#### Conditioning task

##### Data pre-processing and exclusions

All trials with RTs under 100 ms (suggesting automatic, rather than deliberate, responding) and greater than 3SD from each subject’s mean were excluded. This resulted in removal of 373 trials (4.57% of all trials). Subjects missing over 25% of trials were marked for exclusion. No such subjects were identified.

Accuracy data for the conditioning task was then converted into type I *d’*, in order to account for potential response bias. Type I *d’* is a signal detection-theoretic measure of sensitivity to signal versus noise (Stanislaw and Todorov, 1999). While *d’* is not a direct measure of learning, we consider it a good proxy—if participants successfully learn the stimulus-action associations, they should have a greater discrimination ability, reflected as greater *d’. D’* was computed for each cue separately using the proportions of correct (correct action deployed for the corresponding cue, e.g. cue A—action A; Hits) and incorrect (wrong action deployed, e.g. cue A—action B or no action; False Alarm) responses.

##### Evidence of conditioning

In order to assess the presence of learning, one-sample *t*-tests were used to contrast the *d’* values for each cue type against 0 (indicating no ability to discern signal from noise). By proxy, a *d’* of 0 can be taken as an indicator that learning failed to take place. Bs were computed with H_1_ modelled as a half-normal distribution centred on 0, with an SD equal to an approximate expected effect size of *d*’ = 1 (corresponding to 70% hit rate). Analysis was conducted in R (R Core Team, 2018).

The average *d*’ scores for all cues throughout the task were significantly above 0 (Table 1), suggesting that learning of the cue-action association was successfully established.In the conditioning stage, subjects were marked as ‘learners’ if their *d’* scores for both cues *A* and *B* in block 2 (where learning should be evident if it had taken place) were greater than 0. The no-action cue was not included in the learning calculation because the presence of a coloured fixation dot indicating no action was required made the response obvious. Indeed it yielded a nearly perfect accuracy and nearly perfect hit rates with very few false alarms, and this occurred regardless of the ability to learn the associations between the other cues and their outcomes. This near ceiling level performance resulted in an extremely high no-action cue *d’;* although it is noteworthy that d’ is in principle unbounded. Fifty-five (out of 68) subjects were identified as learners and included in the next analysis stage.

**Table 1.** Mean type I *d*’ and SE for each cue type in both blocks, and total, in the conscious conditioning task (Exp.1)

	Conscious conditioning											
Cue	Task total				Block 1				Block2			
	***d’***	***SE***	***P***	***B*_H(0,1)_**	***d’***	***SE***	***P***	***B* _H(0,1)_**	***d’***	***SE***	***P***	***B*_H(0,1)_**
Cue A	1.58***^+^	0.21	<0.001	>10^10^	1.01***^+^	0.22	<0.001	>10^10^	2.46***^+^	0.25	<0.001	>10^10^
Cue B	1.50***^+^	0.23	<0.001	>10^10^	0.67*^+^	0.26	0.011	>10^10^	2.71***^+^	0.27	<0.001	>10^10^
No-action	4.46***^+^	0.06	<0.001	Inf	4.43***^+^	0.08	<0.001	Inf	4.53***^+^	0.05	<0.001	Inf

Notes: Stars indicate significant difference from 0 (*: *P* < 0.05, ***: *P* < 0.001). Cross indicates a sensitive B favouring H1 (+: BH(0,1) > 3), *N* = 68.

#### Breakthrough task

##### Data pre-processing and exclusions

No trials with response times under 100 ms were identified. Unlike the conditioning task, no upper cut-off on RTs was applied, as long response times were expected. All trials where subjects made an incorrect line discrimination (horizontal/vertical) were excluded in order to reduce premature responses and ensure only trials where participants paid attention were analysed (see Discussion for consideration of the related issue of accurately guessed responses). This resulted in the removal of 65 trials (11.96%). Five subjects with 50% (4) or more missing trials were removed. While in the pre-registration we did not expect an upper cut-off would be necessary, one extra trial was removed due to the participant failing to engage with the task, which resulted in a response time of almost 4 min. One subject was removed from both conditions due to a disruption in the testing session. Both cases were noted by the experimenter in the session log. Only the subjects identified as learners in the conditioning stage were brought into the breakthrough time analysis stage. This resulted in a final sample of 49. We confirmed that, averaging across trial types, there was no significant difference in response times between actions A and B (*M*_actionA_ = 5817.87, *M*_actionB_ = 5894.63 ms; *t*(172) = –0.52, *P *=* *0.607).

Each breakthrough trial was given a label describing its cue-action congruence status (i.e. whether the action prepared to indicate breakthrough was congruent or incongruent with the cue, as established in the conditioning task). Action A—cue A and action B—cue B pairs were labelled as congruent pairs. Action A—cue B and action B—cue A were labelled as incongruent pairs. Cue C was always labelled as no-action, and the fourth, previously unseen cue, was labelled novel.

##### Breakthrough time results

Due to the unevenly distributed missing values (i.e. exclusions due to incorrect line orientation judgments) across few data points per participant, the pre-registered analysis method (repeated-measures analysis of variance) was rendered inappropriate. An ANOVA excludes such cases listwise, resulting in excluded participants and reduced power. Given superior performance in treatment of repeated-measures data and data with unevenly distributed missing values, as well as superior ability to model repeated-measures, a generalized linear mixed model (GLMM) was fitted instead. Analysis was conducted using the lme4 package (Bates *et al.*, 2015) in R (R Core Team, 2018).

The model included the raw times of breakthrough as the response variable, and cue-action congruence status (4 levels: congruent, incongruent, no-action, novel) as a fixed effect. We began with a maximal random effects specification (as outlined in the pre-registration), but followed the advice to suppress random slopes due to model non-convergence, retaining subject-specific random intercepts [In R notation, the fixed and random effects of the model were specified as: breakthrough time ∼ congruence index + (1|subjectID)]. (Matuschek *et al.*, 2017; Singmann and Kellen, 2019). A gamma distribution of the response variable, with an identity link function, was specified in order to approximate the nature of response time data without the need for transformations (Lo and Andrews, 2015). The model was fitted by maximum likelihood estimation. All following comparisons were conducted on that model.

The GLMM revealed a significant main effect of congruence status on time of breakthrough (χ^2^ (3) = 12.52, *P *=* *0.006; see Table 2 for regression coefficients). Subsequent pairwise comparisons on estimated means (Tukey-adjusted for multiple comparisons; Table 3, Fig. 3) showed that only the novel, previously unseen cue (*M *=* *6207 ms, *SE* = 64.39) resulted in significantly shorter breakthrough time than both congruent (*M *=* *6311 ms, *SE* = 50.09) and incongruent cues (*M *=* *6356 ms, *SE* = 62.44). Despite a marginally shorter breakthrough time for congruent than incongruent cues, given the adopted priors and the size of the observed effect, the Bayes factor indicates strong evidence against a genuine difference. For B calculation, H_1_ was modelled as a normal distribution centred on 0, with an SD equal to an estimated effect size of 774 ms. This estimate was derived from the observed difference between rewarded and unrewarded cues in a similar b-CFS task conducted earlier by some of the authors (Scott *et al.*, *in preparation*).

**Table 2.** Regression estimates from the GLMM (Exp.1)

	Estimate	*SE*	*t*	*P* (>|z|)
Intercept (congruent)	6311.12	50.09	126.01	< 0.001 **
Incongruent	45.05	30.62	1.47	0.141
No-action	–30.92	36.12	–0.86	0.392
Novel	–103.30	33.93	–3.04	0.002 *

Notes: Congruent cue-action status serves as reference point. Stars indicate significant difference from the intercept (*: *P* < 0.05, **: *P* < 0.001), *N* = 55.

**Table 3.** Pairwise comparisons (tukey-adjusted) of breakthrough time means, estimated in the GLMM (Exp.1)

Congruence status contrast	Estimated mean difference (ms)	*SE*	*df*	*z*-ratio	*P*	*B*_N(0,774)_
Congruent-incongruent	–45.06	30.62	Inf	–1.472	0.459	0.12 ∼
Congruent-no-action	30.92	36.12	Inf	0.86	0.828	0.07 ∼
Congruent-novel	103.30	33.93	Inf	3.04	0.013*	4.48 ^+^
Incongruent-no-action	75.97	48.42	Inf	1.57	0.397	0.21 ∼
Incongruent-novel	148.35	46.24	Inf	3.20	0.008*	10.07 ^+^
No-action-novel	72.38	52.48	Inf	1.38	0.512	0.17 ∼

Notes: Star indicates a significant difference (*: *P* < 0.05). Cross indicates a sensitive B favouring H1 (+: BN(0,774) > 3). Tilde indicates a sensitive B favouring H0 (∼: BN(0,774) < 0.3).

### Conclusions of experiment 1

In Experiment 1, we investigated whether valid sensorimotor predictions, built through instrumental conditioning, can affect conscious experience, operationalized in terms of reportable access to consciousness in breaking interocular suppression. If sensorimotor predictions shape conscious experience, we hypothesised that congruency between the cue and the prepared action would result in the cue breaking through CFS faster.

While the data showed, numerically, marginally shorter breakthrough times for congruent than for incongruent pairs, given the adopted priors and the size of the observed difference the evidence was in favour of the null hypothesis. Specifically, preparing an action congruent with a specific cue (i.e. one previously conditioned with that action) while it was presented under interocular suppression does not shorten the suppression duration relative to preparing an action incongruent with the cue. Therefore, these data do not support the hypothesis that a valid prediction of a cue-action association speeds up reported access to consciousness of the target.

These findings are counter to the results of previous research which did show modulation of perception by action in line with action-congruent percepts (e.g. Maruya *et al.*, 2007; Mitsumatsu, 2009; Beets *et al.*, 2010). While it is possible that our result reflects a genuine absence of influence arising from sensorimotor predictions, it is also possible that the paradigm may have limited the predictive salience of the prepared action. While requiring the same action on each trial within a block ensured counterbalancing of the action-preparation and stimulus congruency, it may also have caused participants to deploy action in an automatic, rather than voluntary, goal-oriented manner. Indeed, previous research has suggested that the stimulus-action contingencies should adaptively reflect a goal or objective in order to facilitate interactions with the world (Hommel *et al.*, 2001, 2010; Prinz, 2003; Mitsumatsu, 2009; Di Pace and Saracini, 2014; Seth, 2014; Seth *et al.*, 2016; Wohlschläger, 2000). The requirement for repetitive action may have inadvertently eliminated this important aspect of the behaviour.

A second potentially confounding influence arises from the timing of the requirement to report the line orientation. Participants were required to press the key corresponding to a vertical or horizontal line overlaid on the stimulus as soon as they began to see the stimulus break through CFS. It is plausible that the decision time relating to the orientation judgment added noise to the measure of breakthrough time. We designed a second experiment, Experiment 2, to address both these issues.

## Experiment 2

Experiment 2 introduced two changes to the breakthrough task described in Experiment 1. Because keeping the action requirement consistent across the entire block may have resulted in participants executing the action in an automatic manner, thus reducing the predictive salience of the prepared action, we varied the action requirement from trial to trial. We also requested the participants to make a single response (with the corresponding action) to indicate that they can see the stimulus, followed by a line orientation judgment performed independently with the other hand. All other task parameters remained the same.

### Materials and Methods

#### Participants

65 participants (18 males; *M* age = 20.78, *SD* = 4.31, range = 18-47) were recruited for the study via the University of Sussex online recruitment system, and an internal mailing list. All participation criteria were identical to Experiment 1. Data for one participant was unusable due to software malfunction, resulting in a sample of 64.

#### Stimuli and materials

All stimuli and materials were identical to Experiment 1.

#### Procedure

The procedure was identical to the conscious conditioning task of Experiment 1 with the exception of the minor changes made to the breakthrough task outlined below.

##### Conditioning task

Identical to Experiment 1.

##### Breakthrough task

The conditioning task was immediately followed by the b-CFS task, using the same set of cues (*cue A, cue B, no-action cue*), as well as fourth, novel cue, never previously seen. The task parameters remained the same as in Experiment 1. In contrast to Experiment 1, the response requirement (action A or B) was no longer repeated without variation within each block. Instead, participants were instructed at trial onset which action would be required to make the response (in a randomized order, but counterbalanced such that each of the four cues is matched with each of the two actions once, resulting in eight trials). This change was considered to be important in order to maintain the predictive salience of the action.

Additionally, in order to eliminate the potentially confounding influence of line orientation judgment on pure breakthrough time, these two responses were separated. Participants were required to respond with the instructed action (A or B) using the return key as soon as they saw the image break through (as opposed to responding as soon as they were able to discriminate the line orientation). After the initial response both the image and the Mondrian pattern disappeared and participants were required to indicate the perceived line orientation; this was done using the ‘1’ (horizontal) and ‘2’ (vertical) buttons at the top of the keyboard using their left hand.

### Results

#### Conditioning task

##### Data pre-processing and exclusions

Pre-processing and exclusion procedures were identical to Experiment 1. All trials with RTs under 100 ms and under or over 3SD from each subject’s mean were excluded. This resulted in removal of 225 trials (2.9% of all trials). Subjects missing over 25% of trials were marked for exclusion. No such subjects were identified.

##### Evidence of conditioning

As in Experiment 1, one-sample t-tests were used to contrast the *d’* values for each cue type against 0 (indicating no sensitivity to signal versus noise). By proxy, a *d’* of 0 can be taken as an indicator that learning failed to take place. Bs were computed with H_1_ modelled as a half-normal distribution centred on 0, with an SD equal to an estimated expected effect size of *d*’ = 1 (corresponding to 70% hit rate).

The *d*’ scores for all cues in both blocks of the conditioning task, and in total, were significantly above 0 (Table 4), suggesting that learning of the cue-action association was again successfully established.

**Table 4.** Mean type I *d*’ and SE for each cue type in both blocks, and total, in the conscious conditioning task (Exp.2)

	Conscious conditioning											
Cue	Task total				Block 1				Block2			
	*d’*	*SE*	*P*	*B*_H(0,1)_	*d’*	*SE*	*P*	*B*_H(0,1)_	*d’*	*SE*	*P*	*B*_H(0,1)_
Cue A	1.54***^+^	0.16	<0.001	>10^10^	0.90***^+^	0.20	<0.001	6632	2.61***^+^	0.20	<0.001	>10^10^
Cue B	1.64***^+^	0.19	<0.001	>10^10^	0.75***^+^	0.24	<0.001	47.17	3.04***^+^	0.23	<0.001	>10^10^
No-action	4.54***^+^	0.07	<0.001	Inf	4.54***^+^	0.08	<0.001	Inf	4.61***^+^	0.07	<0.001	Inf

Notes: Stars indicate significant difference from 0 (***: *P* < 0.001). Cross indicates a sensitive B favouring H1 (+: BH(0,1) > 3), *N* = 64.

Subjects were marked as learners if their *d’* scores for both cues *A* and *B* in block 2 (where learning should be evident if it had taken place) were greater than 0. The *no-action cue* was not included in this criterion, as due to the obvious no-action requirement it yielded a nearly perfect accuracy regardless of the ability to learn the associations between the other cues and their outcomes. Fifty-eight subjects were identified as learners and included in the next analysis stage.

#### Breakthrough task

##### Data pre-processing and exclusions

Pre-processing and exclusion procedures were identical to Experiment 1. No trials with response times under 100 ms were identified. Again, no upper cut-off on RTs was applied, as long response times were expected. All trials where subjects made an incorrect line discrimination were excluded. This resulted in the removal of 46 trials (9%). Three subjects with 50% (4) or more missing trials were removed. This resulted in the final sample of 55 subjects. While in the pre-registration we did not expect an upper cut-off to be necessary, we did encounter five extremely long trials, which were negatively affecting the data distribution (assessed with inspecting the QQ plots) and thus preventing model convergence. Those trials were also removed. Again we confirmed that, averaging across trial types, there was no significant difference in response times between actions A and B (*M*_actionA_ = 6610.41 ms, *M*_actionB_ = 6217.86 ms; *t*(192) = 0.36, *P *=* *0.715).

Each breakthrough trial was given a label describing its cue-action congruence status following the identical procedure as used in Experiment 1.

##### Breakthrough time results

The model and analysis performed was identical to that in Experiment 1. A GLMM was fitted to the data, including the raw times of breakthrough as the response variable. Model specification included the cue-action congruence status (four levels: congruent, incongruent, no-action, novel) as a fixed effect, and subject-specific random intercepts, and was fit with a gamma distribution with an identity link function (Lo and Andrews, 2015). The model was fitted by maximum likelihood estimation. All following comparisons were conducted on that model.

As in Experiment 1, the GLMM revealed a significant main effect of congruence status on time of breakthrough (χ^2^ (3) = 158.28, *P *<* *0.001; see Table 5 for regression coefficients). Subsequent pairwise comparisons on estimated means (Tukey-adjusted for multiple comparisons; Table 6, Fig. 3) showed that cue-action congruent breakthrough times (*M *=* *6516 ms, *SE* = 30.3) were significantly shorter than incongruent breakthrough times (*M *=* *6809 ms, *SE* = 44.6), mirroring the direction of effect found in Experiment 1. Crucially, however, in this instance the size of the difference was substantial, with the Bayes factor providing strong evidence in favour of H_1_. Cue-action congruent breakthrough times were also shorter than no-action breakthrough times (*M *=* *6691 ms, *SE* = 39.6), but significantly longer than breakthrough times for novel cues (*M *=* *6340 ms, *SE* = 41.9; Table 6 for pairwise comparisons). In addition, the cue-action incongruent breakthrough times were significantly longer than no-action breakthrough times. Novel cues resulted in the shortest breakthrough times.

**Table 5.** Regression estimates from the GLMM (Exp.2)

	Estimate	*SE*	*t*	*P* (>|z|)
Intercept (congruent)	6516.27	30.34	214.80	<0.001***
Incongruent	292.94	34.47	8.50	<0.001***
No-action	174.73	23.52	7.43	<0.001***
Novel	–176.47	28.88	–6.111	<0.001***

Notes: Congruent cue-action status serves as reference point. Stars indicate significant difference from the intercept (*: *P* < 0.05, **: *P* < 0.001), *N* = 49, *N* of observations: 412.

**Table 6.** Pairwise comparisons (tukey-adjusted) of breakthrough time means, estimated in the GLMM (Exp.2)

Congruence status contrast	Estimated mean difference (ms)	*SE*	*df*	*z*-ratio	*P*	*B*_N(0,774)_	*meta-B* _N(0,774)_
Congruent-incongruent	–293	34.5	Inf	–8.50	<0.001***	>10^10+^	>10^7+^
Congruent-no-action	–175	23.5	Inf	–7.43	<0.001***	>10^10+^	>10^7+^
Congruent-novel	176	28.9	Inf	6.11	<0.001***	>10^6+^	>10^6+^
Incongruent-no-action	118	41.5	Inf	2.85	0.023 *	3.13^+^	6.63^+^
Incongruent-novel	469	44.6	Inf	10.53	<0.001***	>10^10+^	>10^10+^
No-action-novel	351	38.7	Inf	9.06	<0.001***	>10^10+^	>10^10+^

Notes: Star indicates a significant difference (*: P <* *0.05, ***: *P *<* *0.001). Cross indicates a sensitive B favouring H_1_ (^+^: B_N(0,774)_ > 3). The final column presents meta‐analytic Bayes factors, obtained by pooling the parameters from experiments 1 and 2.

The statistics obtained in pairwise comparisons for Experiment 1 and 2 were then combined in order to calculate a meta-analytic Bayes factor, a single Bayes factor indicating evidence for H_0_ or H_1_ in a group of studies. Posterior parameters were computed for each pairwise comparison using the estimated mean differences and standard errors from Experiment 1 as priors, and the estimated mean differences and standard errors from Experiment 2 as likelihoods (following the method from Dienes, 2014). The resulting mean and the standard deviation of the posterior distribution were then used in the meta-B calculation in a manner identical to regular B calculation, where H_1_ was modelled as a normal distribution centred on 0, with an SD equal to an estimated effect size of 774 ms. See Table 6 for the meta-Bs for each cue-action pairing.

### Conclusions of experiment 2

In Experiment 2, we continued to investigate whether sensorimotor predictions, built through instrumental conditioning, can affect conscious experience, as measured by reported access to consciousness in breaking interocular suppression. We amended the b-CFS task used in Experiment 1 in order to address limitations in the initial design. If sensorimotor predictions shape reported conscious experience, we predicted that congruency between the prepared action and the cue would cause the cue to break through CFS faster.

The results show that a valid sensorimotor prediction does affect the speed of reported access to consciousness. Preparing an action that was congruent with the cue it was conditioned on resulted in significantly faster breakthrough than an incongruent action. This finding is further supported by the meta-analytic Bayes factor analysis, which pooled the results from Experiments 1 and 2. Together the data provides strong support for the hypothesis that conscious experience is affected by sensorimotor predictions—if the executed action is congruent with the cue-action predictive model, reported access to consciousness is facilitated. While the central hypothesis was supported, the shortest breakthrough time was seen for the novel cue. While not in conflict with the key finding, the framework of action-oriented predictive processing does not offer a ready explanation for this pattern. We return to this issue in the general discussion.

## General Discussion

Predictive processing proposes that conscious experience is shaped or constituted by the predictive model that best explains the incoming sensory input. Action-oriented interpretations expand this notion, suggesting that perceptual predictions should encompass sensorimotor contingencies—i.e. predictions of the relationships between an agent’s actions and changes in the world (Seth, 2014, 2015; Clark, 2015, 2016). We sought to empirically test whether conscious experience is affected by valid predictions about learned sensorimotor (stimulus-action) associations—which we here call ‘sensorimotor predictions’.

We developed a novel paradigm in which we used instrumental conditioning to build arbitrary, contextual sensorimotor associations, linking distinct cues with specific actions or with no action. Conditioning was followed by a breaking-CFS task, where a high-contrast pattern was used to suppress visual awareness of the target cues while participants prepared to respond with cue-congruent or non-congruent actions. Through the proxy of breaking interocular suppression, this paradigm allowed us to test the extent to which maintaining the relevant sensorimotor prediction through action preparation facilitates reported conscious access to the associated stimulus, relative to non-congruent stimuli, as well as stimuli associated with no action, and novel stimuli that had not undergone any conditioning exposure.

Experiment 1 failed to find a relationship between sensorimotor predictions and breakthrough times. This finding went against our initial hypothesis and conflicted with previous evidence showing modulation of conscious perception by action (Maruya *et al.*, 2007; Mitsumatsu, 2009; Beets *et al.*, 2010; Di Pace and Saracini, 2014). However, the design of Experiment 1 likely limited the predictive salience of the prepared action. Requiring the same action on each trial within a block (targeted at counterbalancing the stimulus-action congruency) may have caused participants to deploy action in an automatic, rather than dynamic, goal-oriented fashion that diminished the relative importance of action related predictions. It is also noteworthy that with a relatively small number of training trials, sleep might be an important factor in consolidating the learned sensorimotor predictions (e.g. Hindy *et al.*, 2019). While here we chose to focus on methodological changes to sensitivity of the task, this could be a potential avenue for future research.

In Experiment 2, we addressed this limitation by varying the action requirement on each trial. We also reduced the potential for measurement noise in the breakthrough judgment by separating the response indicating the moment of conscious breakthrough from the response indicating the line orientation. While both experiments delivered numerically shorter breakthrough times for congruent versus incongruent cue-action pairings, the modifications designed to improve the predictive salience of the action resulted in considerably larger differences. In Experiment 2, Bayes factor analysis showed strong evidence in favour of a genuine difference based on congruency. Breakthrough time for the no-action cue was also longer than for the action-congruent cue. This is again consistent with our hypothesis, because executing either action A or B would engage the corresponding prediction related to perception of cue A or B, effectively rendering the no-action cue incongruent with either. Importantly, a meta-analytic Bayes factor analysis, where the results from Experiments 1 and 2 were pooled to obtain a single Bayes factor for each comparison, provides strong support for the alternative hypothesis (i.e. congruent predictions accelerate conscious access) across the two experiments.

This result supports the action-oriented interpretations of predictive processing, where conscious experience is affected by valid sensorimotor predictions. It extends the past evidence of a facilitatory role of action on perception (Maruya *et al.*, 2007; Mitsumatsu, 2009; Beets *et al.*, 2010; Dogge *et al.*, 2019; Suzuki *et al.*, 2019) by showing that learned contextual associations can furnish sensorimotor predictions to enhance perception. This result stands in contrast to the ‘cancellation’ accounts, where expected events, such as the consequences of one’s movements, are tuned down and perceived less (Cardoso-Leite *et al.*, 2010). In a recent series of studies, Yon and colleagues (2018, 2020) empirically contrasted the ‘enhancement’ and ‘cancellation’ models, showing that action-congruence biases participants’ perception towards expected outcomes and sharpens the expected sensory representations, in line with Bayesian inference (see also Schwarz *et al.*, 2018; Yon et al., 2020a,b). Our result provides support for this conclusion, and could rely on a similar mechanism.

A surprising finding in both Experiments was that the novel cue was significantly faster to break through than both the congruent and incongruent cue-pairings. While this result is contrary to some previous research examining the facilitatory effects of expectation on access to awareness (e.g. Pinto *et al.*, 2015), it is consistent with reports of greater attentional capture for the first unannounced presentation of novel cues (Al-Aidroos *et al.*, 2010; Becker and Horstmann, 2011; c.f. Meijs *et al.*, 2018a; Ernst *et al.*, 2020). Under predictive processing, these effects might seem at odds—presentation of a novel, unexpected cue yields a high PE, which in turn should take longer to be resolved, resulting in slower, rather than facilitated, perception. Indeed, there is an active debate in the predictive processing literature about whether predictions (expectations) or PEs (incoming sensory signal) should dominate perceptual content and perceptual access. The balance is proposed to be modulated by attention, which up- or down-weights precision afforded to predictions or PEs (Feldman and Friston, 2010; Hohwy, 2012). Recently, it has been proposed that perception is initially biased towards the expected, but particularly salient or surprising events—such as a novel, unpredicted stimulus in our study—are up-weighted (Press *et al.*, 2020). Future extensions of our paradigm may usefully target this question.

It is noteworthy that in the present task we operationalized sensorimotor contingencies as learned arbitrary associations between visual stimuli and subsequent actions. We called those associations ‘sensorimotor predictions’, and manipulated their predictive validity to evaluate reported conscious access. While this mapping constitutes a kind of contingency, one might note that it reverses the direction of what is typically understood as sensorimotor contingencies, i.e. how the world changes in response to our actions (e.g. Seth, 2014). Nor do our learned associations correspond to temporally extended relationships between actions and sensory signals, such as when an object is rotated (e.g. Suzuki *et al.*, 2019). As such, the sensorimotor predictions used here do not correspond to fully-fledged sensorimotor contingencies, but rather to simpler sensorimotor mappings. Nonetheless, the present paradigm could be fruitfully extended to investigate sensorimotor contingencies in the more traditional sense.

The finding that such simple sensorimotor mappings acquired in the learning phase were able to affect visual perception also has broader implications for research in action learning. There has been previous evidence that agents cannot acquire action-outcome predictions to drive intentional action from simple stimulus-response learning, such as when action is cued by a stimulus, as was the case in our paradigm (Herwig *et al.*, 2007; Herwig and Waszak, 2009). However, our result suggests that agents can indeed translate the experience acquired through stimulus-response learning to furnish predictive action-outcome models (in line with Elsner and Hommel, 2001, 2004). While in our study action in the testing phase (bCFS) was forced rather than intentional, investigating whether a similar result is obtained when participants are free to respond intentionally may be an interesting future extension.

An important criticism of b-CFS that should be noted is its interpretation in relation to ‘conscious access’. In b-CFS, the moment understood as the moment of access to consciousness is in fact the moment participants *report* having conscious awareness of the stimulus. Those events may or may not be the same. For example, participants might have rudimentary awareness of the stimulus but delay reporting it (e.g. due to low confidence). As such, our paradigm and many similar paradigms use b-CFS as a proxy measure of speed of access to consciousness, while recognizing this caveat.

Another general criticism of b-CFS is that the time of breakthrough measure may be affected by other processes or biases (Stein and Sterzer, 2014; Sterzer *et al.*, 2014). A challenge pertaining to all

b-CFS experiments is the difficulty of disentangling the speed of access to conscious (reportable) awareness from the time needed to prepare the response—the measured time of breakthrough inherently includes the time taken to respond, which itself could be affected by other conscious or pre-conscious processes. Our effect could have been driven by unconscious motor priming, where the presence of a masked cue primes a congruent response (e.g. a masked left-pointing arrow priming a participant to respond with a left arrow press; Koivisto and Grassini, 2018; Valuch and Mattler, 2019). In the present experiment, this could have taken the form of a masked cue priming a congruent action. However, participants in our study were actively engaged in preparing the desired response according to instructions on each trial. As such, we believe that any unconscious motor-priming effect would likely be minimal compared to the conscious response preparation, which we argue engages the relevant sensorimotor prediction. Indeed, ongoing action preparation was a key part of our design. Nonetheless, we cannot dismiss the possibility that the effect could be partly driven by motor congruence after breakthrough has spontaneously occurred, in a sense that a cue would speed up the congruent action, relative to the incongruent one. Similarly, a match between the stimulus and the action could increase perceptual confidence, and speed up the response without altering the time of conscious perception. While this is a valid concern, it has been recently shown that higher confidence accompanies both stimulus-congruent and -incongruent actions, relative to neutral actions (Gajdos *et al.*, 2019; Siedlecka *et al.*, 2019). This suggests that perceptual confidence should have a limited effect in our study, at least for the congruent-incongruent comparison.

The time taken to respond could also vary between stimuli or types of response. In this study, the two different responses (index and little finger button presses) were a core feature of our design—indeed, we argued that the preparation of a congruent versus incongruent action is what causes the stimulus to break through faster, in line with the idea that valid sensorimotor predictions shape conscious experience. Importantly, however, average response times (irrespective of congruence) did not differ between the two actions, allowing us to rule out the possibility that any underlying difference in time taken to respond with either action contributed to the result. Nonetheless, this does not preclude the possibility of post-perceptual biases affecting the total response time, such as stimulus features slowing down the action. We attempted to guard against this by ensuring the stimuli were as similar as possible. We used four 90^°^ rotations of the same shape, and (in Experiment 2) disentangled the response indicating the stimulus breakthrough from the response indicating line orientation. Still, however, the extent to which post-perceptual biases affect b-CFS remains a topic deserving further study.

Our b-CFS task used only one presentation per cue-action pairing (e.g. action A was paired once with cue A, cue B, no-action cue, and the novel cue). This design was adopted because we felt that to best address the question of how sensorimotor predictions affect conscious experience, we should focus on the very first conscious experience of the action-associated stimulus. While some previous b-CFS experiments have used multiple trials (e.g. Salomon *et al.*, 2013), repeated exposure itself tends to reduce breakthrough times, which could reduce the ability to observe the main effect of interest. Adopting a single trial per cue-action pairing avoids this issue albeit at the cost of the reduced statistical power resulting from a smaller number of trials. Fortunately, our mixed-effects model proved robust to the small number of observations per participant and converged without issue. In addition, the Bayes Factor calculation ensured that the evidence for or against the differences in breakthrough times was sensitive.

The inclusion of a line orientation judgment was intended to reduce the occurrence of premature responding (i.e. responding prior to conscious perception), and to identify instances where participants’ attention had been suboptimal. We excluded from analysis any trials where the line orientation judgement was incorrect. However, theoretically this still leaves open the possibility that the analysed trials contained some instances where participants responded prematurely but correctly guessed the line orientation; any such trials would contaminate the RTs as a pure indicator of conscious breakthrough. Given that the line orientation decisions were not accompanied by confidence judgments, it is not possible to directly identify accurately guessed responses. However, if guessing of the line orientation was a common occurrence, then we would reasonably expect the accuracy of the line orientation judgements to be substantially reduced. Given the observed accuracy was 88 and 91% in Experiments 1 and 2, respectively, we feel reassured that the potential contribution from accurate guesses is likely to have been negligible.

In conclusion, in two experiments we investigated the effect of valid sensorimotor predictions on conscious experience, measured through breaking interocular suppression. The combined data from the two experiments provides strong evidence that preparing a cue-congruent action results in more rapid reported conscious perception of the suppressed stimuli. This provides evidence for a key theoretical implication of the action-oriented predictive processing approach to conscious perception, namely, that sensorimotor predictions shape conscious experience of the world.

## Supplementary Material

niab003_Supplementary_Data
