# Supplementary material for: Sensorimotor predictions shape reported conscious visual experience in a breaking continuous flash suppression task
Source: Neurosci Conscious. 2021 Mar 18;2021(1):niab003. doi: 10.1093/nc/niab003 (PMC7970722; doi:10.1093/nc/niab003)
Supplement: niab003_Supplementary_Data [file niab003_supplementary_data.docx]

**SUPPLEMENTARY MATERIAL**

**Sensorimotor predictions shape reported conscious visual experience
in a breaking Continuous Flash Suppression task.**

Lina I. Skora ^a,c^, Anil K. Seth ^b,c,d^, & Ryan B. Scott ^a,c^

^a^ School of Psychology, University of Sussex, Brighton, UK, BN1 9RH, UK

^b^ School of Informatics, University of Sussex, Brighton, UK, BN1 9QJ, UK

^c^ Sackler Centre for Consciousness Science, University of Sussex, Brighton, UK, BN1 9QJ, UK

^d^ Canadian Institute for Advanced Research, Program on Brain, Mind and Consciousness, Toronto, Canada

**Experiment 1: Unconscious condition**

1. Method
   1. *Participants*

As reported in Experiment 1 described in the paper.

- 1. *Stimuli*

As reported in Experiment 1 described in the paper.

- 1. *Procedure*
     1. Unconscious conditioning.

The conditioning task was used to establish the sensorimotor (stimulus-action) associations. The task used stimuli selected from the assigned set of four. One of the stimuli (*cue A*) was paired with action A (an index finger button press), a second (*cue B*) with action B (a little finger button press), and a third (*no-action cue*) with no action. For practical reasons, the index finger button press was made on the left arrow, and the little finger button press on the right arrow. Note, while the keys being pressed differed, we consider the finger used to make the button press to be the conditioned action. Cues were randomly assigned to a given type for each participant. The order or presentation was randomised.

In the unconscious condition, stimuli were presented under CFS (Tsuchiya & Koch, 2005), in a mirror stereoscope setup. The dominant eye received a Mondrian pattern composed of coloured rectangles, flashing at a rate of 10Hz. The non-dominant eye received the target stimulus. Ocular dominance was established prior to beginning with a standard Miles test (Miles, 1930). Three cues selected from the assigned set of four were presented subliminally using CFS, in a randomised order.

The unconscious presentation of each cue started with a fade-up period of 500ms. Further 500ms after the cue reached full contrast, the fixation dot (overlaid on displays to both eyes) changed colour, indicating that a response was required (see *Figure 1* for an illustration of trial sequence). Fixation dot colours provided the distinction between action and no-action trials. For action cues, participants were instructed to respond with either action A or B (of their choosing), when prompted by the fixation dot changing colour from white to black. Following the action, positive or negative reinforcing feedback (‘correct!’ or ‘wrong!’ printed on the screen, and a cash register or buzz sound, respectively) was delivered, depending on the correspondence of the executed action to the cue presented, thus instantiating instrumental conditioning (e.g. if *cue A* is presented and *action A* is executed, positive feedback is delivered, but if *action B* is executed, negative feedback is delivered). For non-action cues, the fixation dot started as blue and changed colour to red. This indicated to participants at the onset of the trial that no response will be required. As such, the subliminally presented cue became associated with the ‘no action’ response. Participants performed 120 conditioning trials in two blocks of 60, with a 1 minute break between the blocks.

On action cue trials, the cue then disappeared as soon as the participant made a response (1000ms + reaction time after cue onset). This ensured that the action was prepared and executed while the participant was exposed to the cue, but exposure was not prolonged unnecessarily following the response. On no-action trials, the cue disappeared as soon as the fixation dot changed (1500ms after cue onset). This additional 500ms sought to roughly equalise the total exposure duration for no-action trials, where there was no response time, and action trials where the exposure extended to include the participant’s response time.


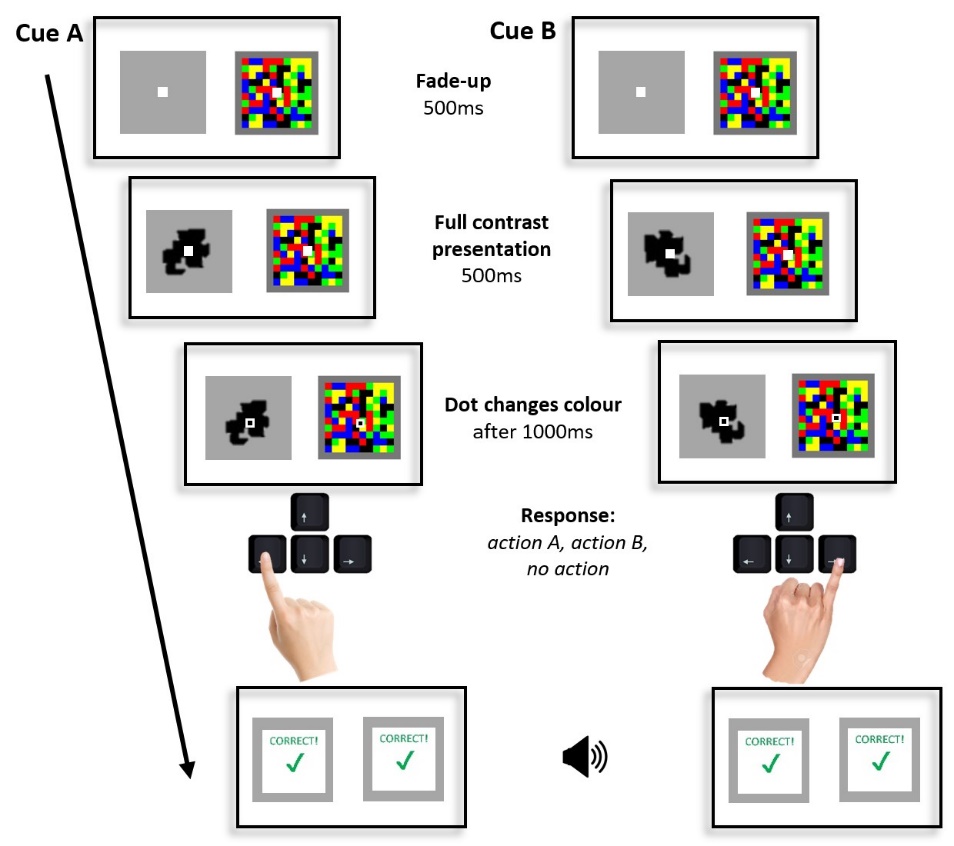


*Figure S1.* Unconscious instrumental conditioning task. Chronological screenshots depict a single trial sequence for two action cues – each panel shows the images shown to the left and right eyes. After 1000ms (including 500ms fade-up of the cue), the fixation dot changes colour from white to black (with a white border), participants can execute the desired action (A, an index finger button press on the left arrow, or B, a little finger button press on the right arrow). For a no-action cue, the fixation dot would change colour from red to blue. Here, the action executed corresponded to the cue type, and the participant was rewarded with visual and auditory feedback.

- - 1. Breakthrough task.

The breaking CFS task in the unconscious condition was identical to the breaking CFS task in the conscious condition, as described in Experiment 1 in the main paper.


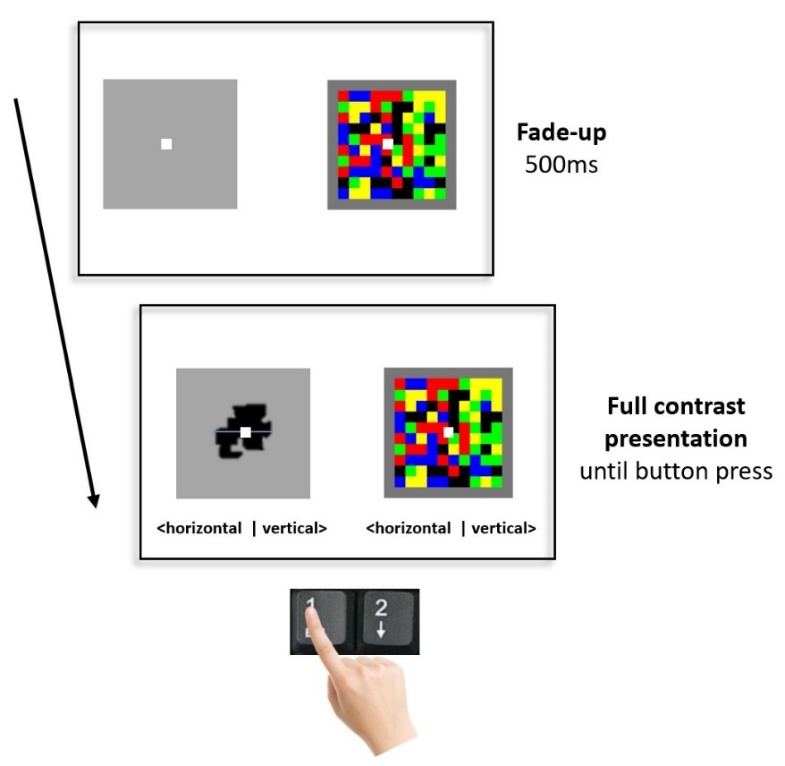


*Figure S2.* Breaking-CFS task. Screenshots depict a single trial sequence (identical for all cues). Following 500ms fade-up, the cue remained on-screen with a horizontal or vertical line overlaid on top of it. Participants were requested to make a response indicating the observed line orientation as soon as they could. This response was to be made with either their index finger or little finger depending on the action randomly assigned to that block. On a given trial, the cue presented could be congruent with the trained action (i.e. associated with it in the conditioning stage), incongruent with it, associated with no action, or novel (not associated with any action).

- - 1. Awareness check.

The session ended with an awareness check, which sought to verify that the cues presented under CFS in the unconscious conditioning task were outside of conscious awareness. The task included one block of 60 trials, and was identical to the unconscious conditioning task, with the exception of the cues, which were two distinct symbols: one black rectangle, and one irregular shape resembling the shapes used in the main task. The symbols were randomly allocated to be rewarding or punishing for each participant, and their order was randomised. There was no no-action cue. Following the feedback, participants were asked to judge if the cue presented under CFS was symmetrical (rectangle) or asymmetrical (the other cue). Next, they were asked to assess their confidence on a binary scale (*some confidence* vs *total guess*). Accuracy, confidence, and their correlations were computed to assess participant’s awareness of the cues during the task. Upon completion, participants were thanked and debriefed.

1. Results
   1. *Conditioning task*
      1. Data pre-processing and exclusions for awareness

All trials with RTs under 100ms (suggesting automatic, rather than deliberate, responding) and under/over 3SD from each subject’s mean were excluded. This resulted in removal of 113 trials (1.38%) in the unconscious conditioning task. Subjects missing over 25% of trials were marked for exclusion. No such subjects were identified.

Subjects who showed visual awareness of the cues, assessed by above-chance judgment accuracy and a positive correlation with confidence in the awareness check, were excluded from further analysis. Those who scored below chance on accuracy, and those who were above chance but showed no correlation (0 or below) were retained. This resulted in the exclusion of 37 out of 68 subjects in the unconscious task (31 participants remaining).

- - 1. Evidence of conditioning.

In order to assess the presence of learning one-sample t-tests were used to contrast the d’ values for each cue type against 0 (indicating no sensitivity to signal versus noise). While *d’* is not a direct measure of learning, we consider it a good proxy – if participants successfully learn the stimulus-action associations, they should have a greater discrimination ability, reflected as greater *d’.*  By such proxy, a *d’* of 0 can be taken as an indicator that learning failed to take place. Bayes Factors were computed with H_1_ modelled as a half-normal distribution centred on 0, with an SD equal to approximate expected effect size of *d*’ = 0.7 for the unconscious condition, following past research examining subliminal learning (Pessiglione et al., 2008). Analysis was conducted in R (R Core Team, 2018).

In unconscious conditioning, only the *no-action cue* was significantly above 0 (see Table 1). For the action-related cues, average *d’* for the length of the task was not significantly different from 0, with evidence favouring the null hypothesis, suggesting that subjects were unable to discriminate between the cues, and thus failed to learn the cue-action association. Performance on *cue A* in block 2 was an exception, with an insensitive B. On average, this result demonstrates that unconscious instrumental conditioning was not achieved in the present paradigm.

**Table 1.** Mean type I *d*’ and SE for each cue type in both blocks, and total, in the unconscious conditioning task. Stars indicate significant difference from 0 (**: p* < 0.05, ***: p* < 0.001). Cross indicates a sensitive B favouring H_1_ (^+^: B_H(0,0.7)_ > 3). Tilde indicates a sensitive BF favouring H_0_ (~: B_H(0,0.7)_ < 0.3). N = 31.

| **Cue** | **Task total** | | | | **Block 1** | | | | **Block2** | | | |
| --- | --- | --- | --- | --- | --- | --- | --- | --- | --- | --- | --- | --- |
|  | ***d’*** | ***SE*** | ***p*** | ***B*** | ***d’*** | ***SE*** | ***p*** | ***B*** | ***d’*** | ***SE*** | ***p*** | ***B*** |
| Cue A | -0.06 | 0.21 | 0.80 | 0.23 | -0.25~ | 0.20 | 0.23 | 0.13 | 0.19 | 0.28 | 0.51 | 0.66 |
| Cue B | -0.32*~ | 0.17 | 0.07 | 0.08 | -0.44*~ | 0.19 | 0.03 | 0.08 | -0.20~ | 0.21 | 0.344 | 0.16 |
| No-action | 4.44**^+^ | 0.08 | <0.001 | Inf | 4.56**^+^ | 0.06 | <0.001 | Inf | 4.35**^+^ | 0.11 | <0.001 | Inf |

In the conditioning stage, subjects were marked as ‘learners’ if their *d’* scores for both cues *A* and *B* in block 2 (where learning should be evident if it had taken place) was greater than 0. The no-action cue was not included in the learning calculation because the presence of a coloured fixation dot indicating no action was required made the response obvious. Indeed it yielded a nearly perfect accuracy and nearly perfect hit rates with very few false alarms, and this occurred regardless of the ability to learn the associations between the other cues and their outcomes. This near ceiling level performance resulted in an extremely high no-action cue *d’;* although it is noteworthy that d’ is in principle unbounded.

12 (out of the 31 determined as unaware) subjects were identified as ‘learners’. However, given that learning was absent at the group level, the learners were not passed on to the next analysis stage due to the risks for robustness of the analysis. Firstly, post-hoc selection of the aware subjects from the sample risks obtaining a false-positive through regression to the mean (Shanks, 2017). Secondly, a small sample size was likely to result in insufficient power. As such, analysis for the unconscious conditioning condition of the task was terminated after the conditioning stage. Since we were interested in predictions about learned stimulus-action associations (sensorimotor predictions) on breaking-CFS, it is not informative to analyse data from a condition where these associations were not learned.
